# Intra-Articular Catheter Placement: A Novel Approach for Simulating Ankle Effusions in Cadaver Models

**DOI:** 10.5811/westjem.2018.9.39810

**Published:** 2018-11-13

**Authors:** Graeme A. Ross, Nicholas G. Ashenburg, W. David Wynn, Jordan J. McCarthy, Alexander M. Clendening, Bradley C. Presley, Steven W. Kubalak, Ryan M. Barnes

**Affiliations:** * Medical University of South Carolina College of Medicine, Department of Emergency Medicine, Charleston, South Carolina; † Medical University of South Carolina College of Medicine, Charleston, South Carolina; ‡ Medical University of South Carolina Center for Anatomical Studies and Education, Department of Regenerative Medicine and Cell Biology, Charleston, South Carolina

## BACKGROUND

Arthrocentesis is a clinical procedure employed by a number of medical subspecialties. Two techniques are commonly used for arthrocentesis: landmark and ultrasound. A number of different models exist to teach arthrocentesis including gel, plastic, and cadaveric types. Evidence suggests that cadaver types may be superior.^1^ Cadavers are an excellent model to teach arthrocentesis because their joints can be filled with fluid, creating simulated effusions.^1^–^3^ Creating and maintaining consistent effusions for large groups of students can be challenging. The volume required to create a clear effusion varies with each cadaver, and joint capsules are prone to leakage after numerous procedure attempts.^4^ Inadvertent air infiltration from repeated filling can create artifacts that limit visualization by ultrasound. Medical educators seek an arthrocentesis training model that is easily modifiable and can withstand multiple procedure attempts.

## OBJECTIVE

The primary objective of this project was to develop a simple method of creating and maintaining ankle joint effusions in cadavers. Ideally, this method would allow for volume modification and be suitable for both landmark and ultrasound-guided arthrocentesis.

## DESIGN

The method we present here was initially developed to address a problem that we encountered during cadaveric research. We were conducting an ankle arthrocentesis study that required simulated joint effusions. Research personnel experienced difficulty maintaining ankle joint effusions with a single-shot technique. This technique involved temporarily inserting a needle into the joint space and filling it with saline. These simulated effusions tended to leak, inhibiting successful arthrocentesis. We solved this problem by inserting a plastic intravenous (IV) catheter into the joint space. The catheter allowed us to create and maintain a consistent effusion while avoiding interference with arthrocentesis. Recognizing its utility, we adopted this method as the primary means of creating simulated, ankle-joint effusions for this study.

The ideal location for catheter placement is the anterior-lateral joint space as most arthrocentesis techniques involve a more medial approach. We used an ultrasound machine equipped with a linear transducer to help locate the lateral tibiotalar joint space. A 20G 1.75inch (Braun) IV catheter was inserted by ultrasound guidance into the tibiotalar joint. We then connected a short segment of extension tubing (with Luer Lock) to the IV catheter, and both devices were sutured to the skin (Figure). A syringe was used to aspirate free air and any synovial fluid. Using the catheter-tubing setup, 15–20 milliliters of saline was injected directly into the joint. We used ultrasound to confirm a joint effusion and guide size adjustments. Occasionally, fluid would slowly leak from the synovial capsule. This was addressed by slow saline instillation by applying steady pressure to the plunger of a 60cc syringe. The effusion size and consistency were confirmed simultaneously under ultrasound.

## IMPACT/EFFECTIVENESS

We describe a method to create simulated, ankle-joint effusions. Its effectiveness was demonstrated in an ongoing research project. All ankles were filled using this method and each effusion was confirmed by ultrasound. A total of 14 ankles were filled and each ankle joint was aspirated at least once and, in some cases, multiple times. One ankle was aspirated 17 times with no loss of tissue integrity.

Thirty participants attempted both landmark and ultrasound-guided arthrocentesis on these ankles. In total, 60 arthrocentesis procedures were performed. Of the participants, 18 were pre-clinical medical students and 12 were emergency medicine attending physicians. None of the medical students had previously performed an arthrocentesis. Twenty-nine of 30 (97%) participants were successful using both techniques. One participant was unsuccessful in both techniques despite the presence of a confirmed effusion.

Although the participants had varying levels of experience, nearly all were able to complete arthrocentesis using this model. Multiple arthrocentesis attempts were performed successfully without comprising the model’s quality. We think this model has educational benefit especially when teaching large groups. Institutions with limited access to cadavers may be able to use this technique to teach multiple learners. While fresh frozen cadavers may be preferred due to their closer approximation to live-patient tissue qualities, the logistics of having readily available specimens renders this option suboptimal. Obstacles include the high cost of acquisition and subsequent maintenance of the specimens and their relatively short time frame of usefulness once thawed.

Our method of creating simulated joint effusions performed well in embalmed cadavers. Indeed, cadavers have been shown to be a preferred model for teaching arthrocentesis.^1^ We have developed a method that offers a novel way to create and maintain joint effusions in embalmed cadavers while allowing learners with various levels of experience to practice this important procedure.

## Figures and Tables

**Figure:**
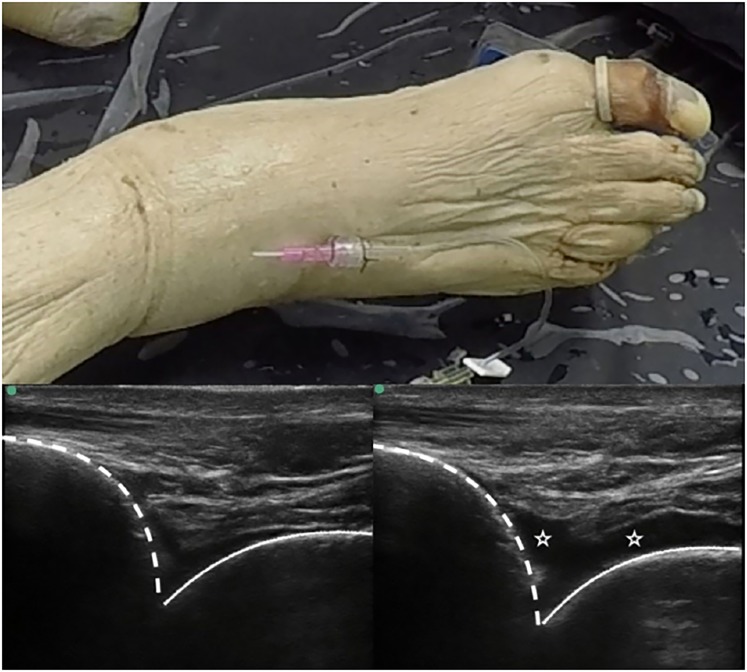
The catheter within the joint used to create effusion. Also shown are examples of an ankle pre- and post- effusion creation. The tibia (dotted line) and talus (solid line), as well as effusion (stars), are shown on ultrasound.
